# Epidemiological investigation, determination of related factors, and spatial-temporal cluster analysis of wild type pseudorabies virus seroprevalence in China during 2022

**DOI:** 10.3389/fvets.2023.1298434

**Published:** 2023-12-04

**Authors:** Wenchao Gao, Xiaoxue Jiang, Zhiqiang Hu, Qing Wang, Yuntong Shi, Xiaogang Tian, Mengli Qiao, Jinyong Zhang, Yang Li, Xiaowen Li

**Affiliations:** ^1^Shandong New Hope Liuhe Co., Ltd., Qingdao, Shandong, China; ^2^Shandong Engineering Laboratory of Pig and Poultry Healthy Breeding and Disease Diagnosis Technology, Qingdao, Shandong, China; ^3^New Hope Liuhe Co., Ltd., Chengdu, Sichuan, China; ^4^Key Laboratory of Feed and Livestock and Poultry Products Quality and Safety Control, Ministry of Agriculture and Rural Affairs, Chengdu, Sichuan, China; ^5^Xiajin New Hope Liuhe Agriculture and Animal Husbandry Co., Ltd., Dezhou, China; ^6^Shandong New Hope Liuhe Agriculture and Animal Husbandry Technology Co., Ltd. (New Hope Liuhe Academy of Swine Research), Dezhou, China

**Keywords:** pseudorabies virus, seroprevalence, epidemiological investigation, related factors, spatial-temporal clustering, China

## Abstract

**Introduction:**

Pseudorabies virus (PRV) is a linear DNA virus with a double-stranded structure, capable of infecting a diverse array of animal species, including humans. This study sought to ascertain the seroprevalence of Pseudorabies Virus (PRV) in China by conducting a comprehensive collection of blood samples from 16 provinces over the course of 2022.

**Methods:**

The presence of PRV gE antibodies was detected through the utilization of an enzyme-linked immunosorbent assay (ELISA) technique. Logistic regression analysis was conducted to identify potential related factors associated with the serologic status of PRV gE at the animal level. Additionally, the SaTScan 10.1 software was used to analyze the spatial and temporal clusters of PRV gE seroprevalence.

**Results:**

A comprehensive collection of 161,880 samples was conducted, encompassing 556 swine farms throughout the country. The analysis revealed that the seroprevalence of PRV gE antibodies was 12.36% (95% confidence interval [CI], 12.20% to 12.52%) at the individual animal level. However, at the swine farm level, the seroprevalence was considerably higher, reaching 46.22% (95% CI, 42.08% to 50.37%). Related factors for PRV infection at the farm level included the geographic distribution of farms and seasonal variables. Moreover, five distinct high seroprevalence clusters of PRV gE were identified across China, with the peak prevalence observed during the months of April through June 2022.

**Conclusion:**

Our findings serve as a valuable addition to existing research on the seroprevalence, related factors, and temporal clustering of PRV gE in China. Furthermore, our study provides a reference point for the development of effective strategies for the prevention and control of pseudorabies and wild virus outbreaks.

## 1 Introduction

Pseudorabies virus (PRV) belongs to the herpesviral subfamily A, which also includes the varicella virus. PRV is the causative agent of pseudorabies (PR), which is more commonly known as Aujeszky's Disease (1). While PRV has the ability to infect various animal species, only pigs serve as the reservoir hosts for this virus (2–7). Upon PRV infection, pigs can exhibit varying clinical symptoms depending on their age at the time of infection. Newborn piglets primarily display neurological symptoms and have high mortality rates, while infected adult sows exhibit reproductive and respiratory disorders (8–10).

Since 2011, there has been a resurgence of PRV in multiple swine farms throughout China. This outbreak is mainly characterized by sow abortion, stillbirth, and increased piglet mortality (11). This resurgence can be attributed to the emergence of PRV variants, such as HN1201, TJ strains, and SDYC-2014 (12–14). Multiple studies have demonstrated that Bartha-K61 deletion strain vaccines are insufficient in providing comprehensive protection against these variants (13, 15).

Despite successful control or eradication of pseudorabies (PR) in many countries through vaccination, the epidemic remains prevalent in Chinese pigs (16). Despite immunization with the PR Bartha-K61 deficiency vaccine, numerous outbreaks of PRV have occurred (13, 17–21). In 2018, there was an outbreak of African swine fever in China, which had a significant impact on the country's swine farming industry. It has led to significant changes in herd distribution, farm biosecurity levels, and herd circulation strategies within the swine farming industry. Zhao et al. found that pig farms sampled after the ASF outbreak demonstrated a lower likelihood of PRV infection compared to those sampled before the outbreak (22). This could be attributed to enhanced biosafety management practices. As a result, the prevalence of PR has been affected (23, 24). There are reports on the serum prevalence of PRV in China before 2021, as well as related factors and spatiotemporal analysis, without relevant data for 2022. Therefore, in this study, more than 160,000 serum samples were collected in China in 2022, its aimed to analyze the current prevalence of pseudorabies and explore the spatiotemporal patterns. Additionally, conducting spatial-temporal analysis of PRV infection can help in identifying clusters with high PRV prevalence and understanding the trends of variation in PRV infection. This information can assist policymakers in designing more precise and cost-effective intervention policies for future PRV control in China.

## 2 Materials and methods

### 2.1 Sample collection

In total, 161,880 serum samples were collected from 556 swine farms belonging to 106 companies across 16 provinces in China in 2022, covering six regions including Northeast China (Heilongjiang Province and Liaoning Province), Central South China (Henan, Hubei, Hunan, and Guangxi), East China (Jiangsu and Shandong), Northwest China (Gansu and Shaanxi), North China (Hebei, Inner Mongolia, Shanxi, and Tianjin), and Southwest China (Guizhou and Sichuan). Furthermore, the location coordinates of pig farms were obtained from Baidu Map (https://map.baidu.com/). The breeding farms, which had a breeding stock ranging from 750 to 3,000 sows, maintained a consistent herd composition. The consistent herd composition of the breeding farms included pre-weaning piglets (piglets that are 0–21 days old), gilts (female pigs that are between 90 and 230 days old), adult sows (over 230 days old, either in gestation or with a history of gestation), and boars (male pigs that are over 300 days old). The pigs in the fattening farm are divided into two categories: nursery piglets and growing-finishing pigs. Nursery piglets were between 21 and 70 days old, while growing-finishing pigs were between 70 and 180 days old. These farms had a production scale of over 6,000 pigs. All adult sows and boars were vaccinated three times a year with live PR vaccines (Bartha-K61 strain) and an inactivated vaccine (Bartha-K61 strain). All fattening pigs on the farms received vaccinations as part of their healthcare routine. At the age of 56 days, they were vaccinated with live PR vaccines. Then, at the age of 84 days, they received vaccinations using inactivated vaccines. For all gilts on the farms, at the age of 119 days, they received vaccinations with live PR vaccines. Subsequently, at the age of 147 days, they were vaccinated with inactivated vaccines. The sampling of pigs was conducted based on the scale and model of breeding. An approximately equal number of samples were collected from various growth stages, including suckling piglets, nursery pigs, fattening pigs, sows, and gilts. This approach ensured a representative distribution of samples across various age groups and production stages, enabling a thorough analysis of the swine being studied. In each season, the sampling frequency varied depending on the size of the farms. Approximately 50–60 serum samples were collected from each small farm (with fewer than 1,000 pigs), 70–100 serum samples from each medium-sized farm (with 1,000–2,000 pigs), and 100–150 serum samples from each large farm (with more than 3,000 pigs). This sampling approach aimed to ensure adequate representation and provide sufficient data for analysis across various farm sizes. All serum samples were collected and stored at −20°C.

### 2.2 Serological detection

Anti-gE antibody levels in serum were quantified using commercially available blocking ELISA Kits (Cat: CP144, IDEXX Laboratories, Westbrook, ME) in accordance with the manufacturer's instructions. Commercial blocking ELISA kits have been specifically developed to distinguish between the vaccine strain and wild strains of PR. In the study, the findings of the blocking ELISA test were reported in terms of sample/negative (S/N) values. A S/N value below 0.60 was deemed positive, suggesting the existence of anti-gE antibodies. Conversely, a S/N value exceeding 0.70 was considered to be indicative of an negative result, suggesting the lack of anti-gE antibodies. S/N values ranging from 0.60 to 0.70 were deemed questionable, necessitating additional testing or repeated testing over a period of time to ascertain the presence or absence of antibodies.

### 2.3 Statistical analysis

In this study, all the gathered data were entered and organized utilizing Microsoft Excel 2021, a widely used spreadsheet software developed by Microsoft in the United States. The associations between the seroprevalence of PRV-gE antibodies and various factors, such as regions, quarters, and pig herds, were analyzed using the logistic regression model in SPSS 26.0 software (IBM, Chicago, IL, USA). The study examined the 95% confidence intervals (CIs) of the results. In this study, statistical significance was determined by a *p*-value of <0.05.

A trend analysis of seroprevalence for PRV gE was conducted by following the steps outlined below. A pig farm was deemed to be positive for wild-type PRV if at least one sample tested positive for gE antibodies. The serological status of a swine farm with regards to PRV was considered as a dichotomous variable, indicating that it was categorized as either PRV-positive or PRV-negative. Spatio-temporal scanning was performed utilizing the Poisson distribution model. The researchers employed SaTScan version 10.1 software to forecast the spatial-temporal distribution of high PRV gE seroprevalence aggregation using the Bernoulli model. The samples obtained from each pig farm were categorized into two groups by the researchers. The first group, referred to as the experimental group, comprised of samples that tested positive for PRV gE antibodies. The second group, known as the control group, consisted of samples that tested negative for PRV gE antibodies. Time aggregation was conducted on a monthly basis, encompassing the entire duration of the experiment, which spanned from January 1, 2022, to December 31, 2022. When the *P*-value of the log-likelihood ratio (LLR) test is <0.05, the region is deemed to demonstrate aggregation. In addition, the creation of maps was facilitated through the utilization of ArcGIS 10.7 software, developed by ESRI, USA.

## 3 Results

### 3.1 Seroprevalence of PRV-gE antibodies

#### 3.1.1 Descriptive statistics of PRV gE seroprevalence

**Figure 1:**
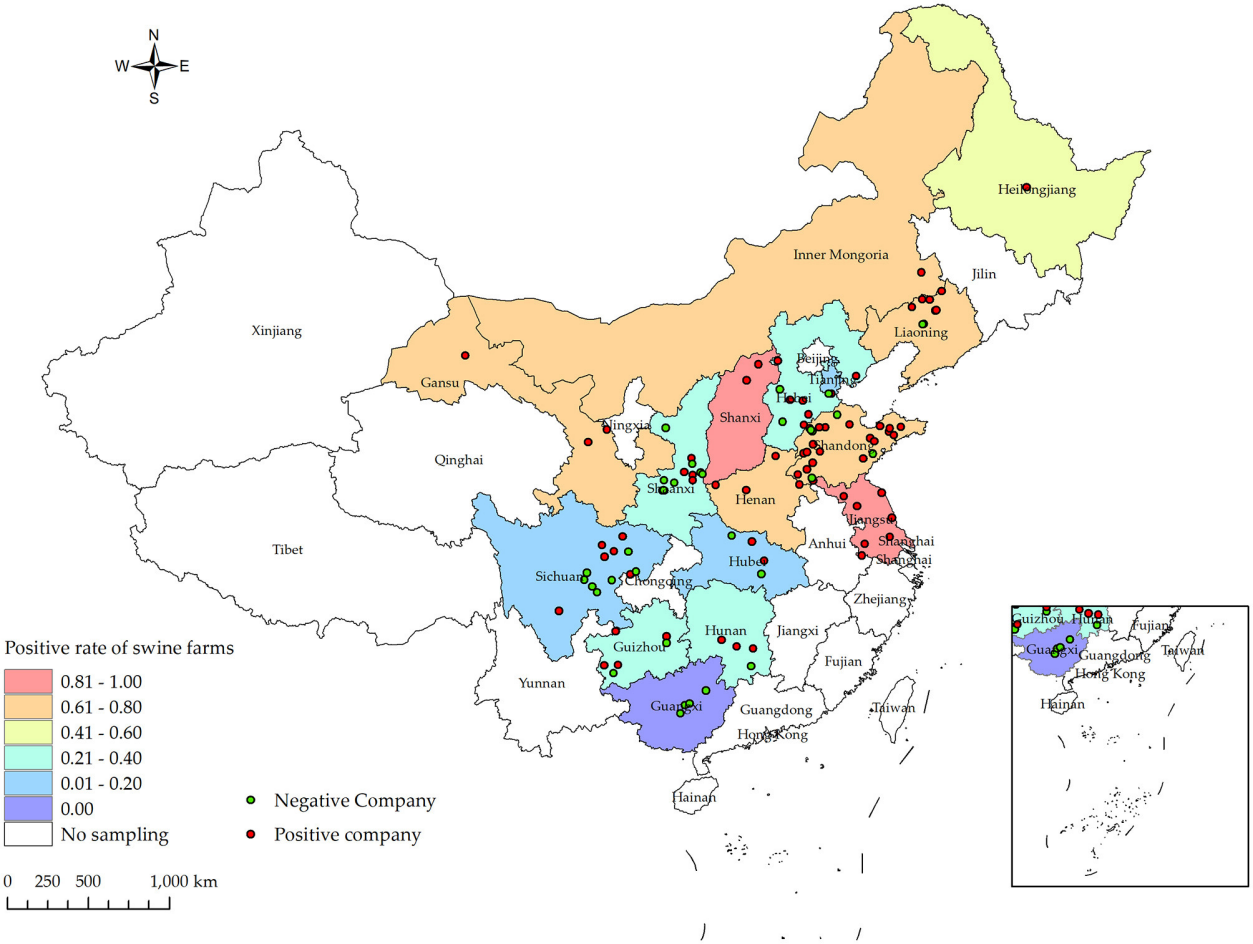
Distribution of PRV gE infection rates at the animal level for samples collected from January 2022 to December 2022 in various provinces and cities in China, along with their respective collection locations. Different shades of box colors represent infection rates, green dots indicate PRV gE antibody-negative companies, and red dots indicate PRV gE antibody-positive companies.

**Figure 2:**
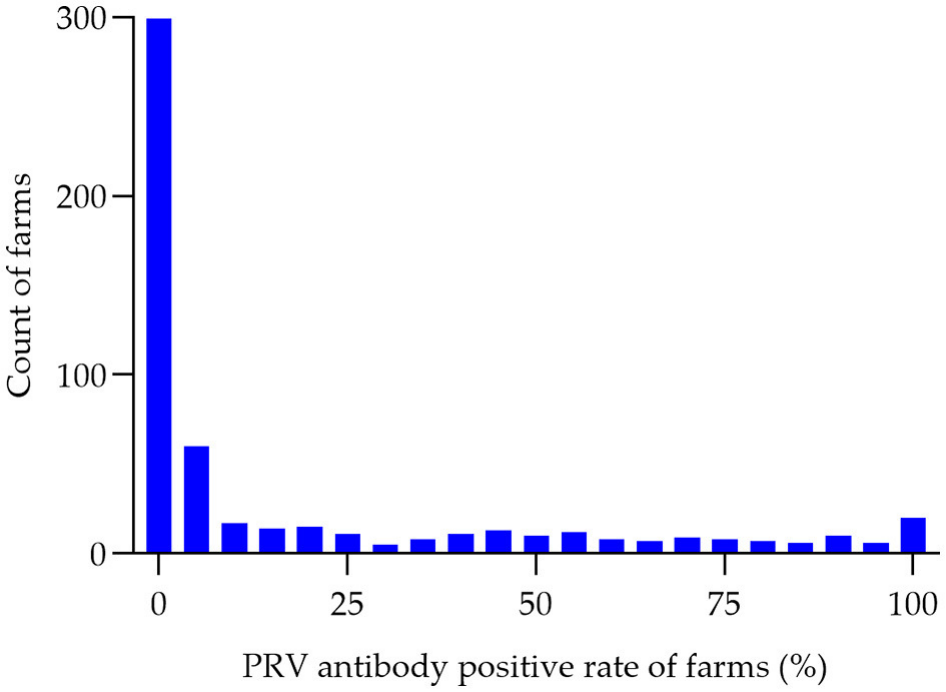
Histogram of PRV antibody-positive rates (%) in pig farms. The seropositivity rate of PRV was determined for each of the 556 swine farms in 2022. The number of swine farms falling within each positivity range was then counted in 5% increments, ranging from 0 to 100%. This data was used to create a histogram, with the horizontal axis representing the positivity range (in groups of 5%) and the vertical axis representing the number of swine farms.

Between the period of January 2022 and December 2022, an extensive collection of 161,880 blood samples was conducted from various sources within China's swine farms. Specifically, the samples were collected from a total of 556 swine farms, which are owned by 106 different companies across 16 provinces (see Figure 1). Out of the total of 556 swine farms surveyed, a significant proportion of 257 farms were found to have tested positive for PRV gE antibodies. According to the data collected in this study, the prevalence of PRV gE seropositivity at the animal level demonstrated significant variation, with a range of 0–100% observed within each farm (Figure 2). The overall prevalence of PRV-gE antibodies in serum samples was found to be 12.36% (20,009/161,880, 95% CI 12.20–12.52%), with a farm positivity rate of 46.22% (257/556, 95% CI 42.08–50.37%) (Table 1).

**Table 1:** Prevalence of PRV gE antibody determined by the Pearson chi-square test in the provinces of China.

**Regular area**	**Province**	**Samples** ^**a**^			**Pig farms** ^**b**^		
		**No. of positive samples**	**Total no. of samples**	**Seroprevalence rate (%) (95% CI)**	**No. of positive farms**	**Total no. of farms**	**Farm positivity rate (%) (95% CI)**
Northeast China	Heilongjiang	2,984	13,747	21.71 (21.02–22.40)	29	60	48.33 (35.69–60.98)
	Liaoning	959	2,129	45.04 (42.93–47.16)	13	17	76.47 (56.31–96.63)
North China	Hebei	251	10,500	2.39 (2.10–2.68)	10	32	31.25 (15.19–47.31)
	Inner Mongolia	974	3,304	29.48 (27.92–31.03)	8	13	61.54 (35.09–87.99)
	Shanxi	896	1,238	72.37 (69.88–74.87)	12	13	92.31 (77.82–106.79)
	Tianjin	6	3,196	0.19 (0.04–0.34)	1	12	8.33 (−7.30–23.97)
East China	Jiangsu	1,430	5,074	28.18 (26.94–29.42)	11	13	84.62 (65.00–104.23)
	Shandong	3,059	26,938	11.36 (10.98–11.73)	77	128	60.16 (51.67–68.64)
Northwest China	Gansu	4,174	16,566	25.20 (24.54–25.86)	30	39	76.92 (63.70–90.15)
	Shaanxi	1,255	26,088	4.81 (4.55–5.07)	21	64	32.81 (21.31–44.32)
Southwest China	Guizhou	488	13,489	3.62 (3.30–3.93)	9	35	25.71 (11.23–40.19)
	Sichuan	109	21,974	0.50 (0.40–0.59)	7	47	14.89 (4.72–25.07)
Central South China	Guangxi	0	1,470	0.00 (-)	0	9	0.00 (-)
	Henan	1,511	4,584	32.96 (31.60–34.32)	18	27	66.67 (48.89–84.45)
	Hubei	1,677	9,843	17.04 (16.29–17.78)	6	34	17.65 (4.83–30.46)
	Hunan	236	1,740	13.56 (11.95–15.17)	5	13	38.46 (12.01–64.91)
Total		20,009	161,880	12.36 (12.20–12.52)	257	556	46.22 (42.08–50.37)

^a^The chi-square test value for PRV gE seropositivity in different provinces under sample conditions was 25,883.38, with a p-value of <0.01.

^b^The chi-square test value for PRV gE seropositivity at the farm level in different provinces was 113.88, with a p-value of <0.01.

#### 3.1.2 Province levels seroprevalence of PRV-gE antibodies

At the animal level, the provinces exhibiting higher rates of PRV gE antibodies are Shanxi, Liaoning, and Henan, with prevalence rates of 72.37% (95% CI, 69.88–74.87%), 45.04% (95% CI, 42.93–47.16%), and 32.96% (95% CI, 31.60–34.32%), respectively. In contrast, the prevalence of PRV gE antibodies in three provinces (Sichuan Province, Tianjin City, and Guangxi Autonomous Region) is found to be <1%. The chi-square test for PRV gE serum numbers indicated a significant difference between the provinces (ranging from 0.00 to 72.37%), with a *P*-value of <0.01 (Table 1). At the farm level, the positivity rates of PRV gE antibodies were highest in Shanxi and Jiangsu provinces, at 92.31% (95% CI, 77.82–106.79%), 84.62% (95% CI, 65.00–104.23%), respectively. In contrast, the lowest rates of PRV gE antibody positivity are 0.00% in the Guangxi provinces. The chi-square test for PRV gE farm numbers indicated a significant difference between the provinces (ranging from 0.00 to 92.31%), with a *P*-value of <0.01 (Table 1).

#### 3.1.3 Region levels of seroprevalence of PRV-gE antibodies

The Northeast region had the highest seroprevalence in serum samples at 24.84% (3,943/15,876, 95% CI 24.16–25.51%). On the other hand, the lowest seroprevalence was observed in the Southwest region at 1.68% (597/35,463, 95% CI 1.55–1.82%). The East region had the highest positive rate of pig farms at 62.41% (88/141, 95% CI 54.42–70.41%). On the other hand, the Southwest region had the lowest positive rate at 19.51% (16/82, 95% CI 10.93–28.09%). Furthermore, significant variations in the positive rates of sera were observed across different regions (chi-square test, *p* < 0.01) (Table 2).

**Table 2:** The Chi-square test of related factors associated with PRV serological status at the sample level.

**Factor**	**Category**	**No. positive**	**No. sample**	**Seroprevalence rate (%) (95% CI)**	***P-*value**
Regions	Northeast China	3,943	15,876	24.84 (24.16–25.51)	Reference
	Central South China	3,424	17,637	19.41 (18.83–20.00)	<0.01
	East China	4,489	32,012	14.02 (13.64–14.40)	<0.01
	Northwest China	5,429	42,654	12.73 (12.41–13.04)	<0.01
	North China	2,127	18,238	11.66 (11.20–12.13)	<0.01
	Southwest China	597	35,463	1.68 (1.55–1.82)	<0.01
Pig herds	Sows	8,659	43,841	19.75 (19.38–20.12)	Reference
	Piglets	2,324	10,629	21.86 (21.08–22.65)	<0.01
	Nursery pigs	1,043	5,675	18.38 (17.37–19.39)	0.01
	Fat pigs	3,617	28,583	12.65 (12.27–13.04)	<0.01
	Gilts	3,968	56,874	6.98 (6.77–7.19)	<0.01
	Boars	398	16,278	2.45 (2.21–2.68)	<0.01
Quarters	Q1	2,539	24,074	10.55 (10.16–10.93)	Reference
	Q2	6,203	42,005	14.77 (14.43–15.11)	<0.01
	Q3	5,395	52,557	10.27 (10.01–10.52)	0.24
	Q4	5,872	43,244	13.58 (13.26–13.90)	<0.01
Total	20,009	161,880	12.36 (12.20–12.52)	

#### 3.1.4 Herd levels seroprevalence of PRV-gE antibodies

**Figure 3:**
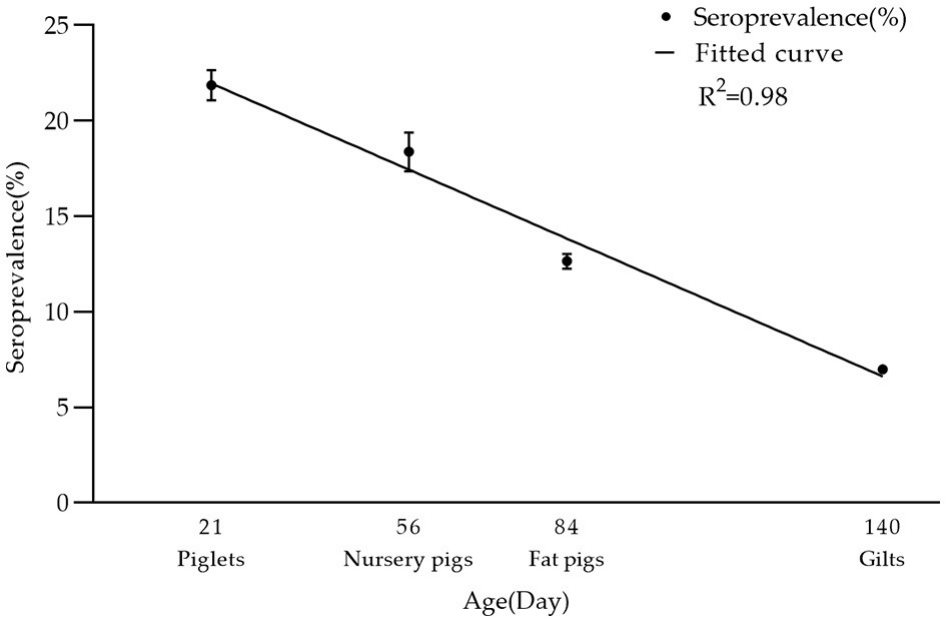
Seroprevalence rate of PRV-gE in different age groups. There was a linear decrease in seropositivity of serum samples from piglets, nursery pigs, fat pigs, to gilts: 21.86% (2,324/10,629, 95% CI 21.08–22.66%), 18.38% (1,043/5,675, 95% CI 17.37–19.39%), 12.65% (3,617/28,583, 95% CI 12.27–13.04%), 6.98% (3,968/56,874, 95% CI 6.77–7.19%). The *R*^2^ value of its trendline is 0.98.

In breeding farms, pigs are categorized into piglets, gilts, sows, and boars based on their growth stages. Fattening farms, on the other hand, house nursery pigs and fattening pigs. As indicated in Table 2, the seroprevalence of serum samples was highest in piglet herds at 21.86% (2,324/10,629, 95% CI 21.08–22.66%), and lowest in boar herds at 2.45% (398/16,278, 95% CI 2.21–2.68%). Furthermore, significant differences were observed between sows and other herds (chi-square test, *p* < 0.05). Interestingly, there was a linear downward trend in seroprevalence from piglets to gilts (Figure 3).

#### 3.1.5 Seasonal levels of PRV-gE antibody seropositivity

In the present investigation, the initial quarter encompasses the months of January, February, and March. The second quarter encompasses the months of April, May, and June. The third quarter encompasses the months of July, August, and September. The fourth quarter, which includes the months of October, November, and December, marks the final period of the year. In terms of quarters, the seropositive rates of the first quarter (Q1) and the third quarter (Q3) were significantly lower than those of the second quarter (Q2) and the fourth quarter (Q4) (chi-square test, *p* < 0.05). Q2 had the highest seropositive rate of 14.77% (6,203/42,005, 95% CI 14.43–15.11%) (Table 2).

### 3.2 Related factor investigation related to PRV serological status

In the chi-square test result (Table 2), it was observed that variables such as regions, pig herds, and quarters had *P*-values of <0.05. Consequently, these variables were included in the multiple logistic regression model. Three related factors associated with the serological status of PRV were identified through multivariate logistic analysis (Table 3). The population density in North China, East China, Northwest, Southwest, and Central South regions is comparatively lower than that in Northeast China. In comparison to the Northeast region, the Southwest region exhibited a significantly lower probability of PRV infection in pigs, with an odds ratio of 0.05 (95% CI, 0.04–0.05). The probability of PRV infection in boars (OR, 0.09; 95% CI, 0.08–0.10), fattening pigs (OR, 0.09; 95% CI, 0.08–0.10), and gilts (OR, 0.32; 95% CI, 0.30–0.33) was significantly lower than in sows. Additionally, the likelihood of PRV infection was found to be higher in Q2, Q3, and Q4 compared to Q1. The probability of PRV infection in pigs during the second quarter is 1.65 times higher (95% CI, 1.57–1.74) compared to the first quarter.

**Table 3:** Multivariable logistic analysis of related factors associated with PRV serological status.

**Factor**	**Category**	**OR (95%CI)**	***P-*value**
Regions	Northeast China	1 (Reference)	
	Central South China	0.54 (−0.51–0.57)	<0.01
	East China	0.33 (0.32–0.35)	<0.01
	Northwest China	0.33 (0.31–0.34)	<0.01
	North China	0.34 (0.32–0.36)	<0.01
	Southwest China	0.05 (0.04–0.05)	<0.01
Pig herds	Sows	1 (Reference)	
	Piglets	1.04 (0.98–1.10)	0.16
	Nursery pigs	0.98 (0.91–1.05)	0.52
	Fat pigs	0.58 (0.55–0.60)	<0.01
	Gilts	0.32 (0.30–0.33)	<0.01
	Boars	0.09 (0.08–0.10)	<0.01
Quarters	Q1	1 (Reference)	
	Q2	1.65 (1.57–1.74)	<0.01
	Q3	1.21 (1.15–1.28)	<0.01
	Q4	1.58 (1.49–1.66)	<0.01

### 3.3 Spatio-temporal clustering investigation of high seroprevalence of PRV gE

**Figure 4:**
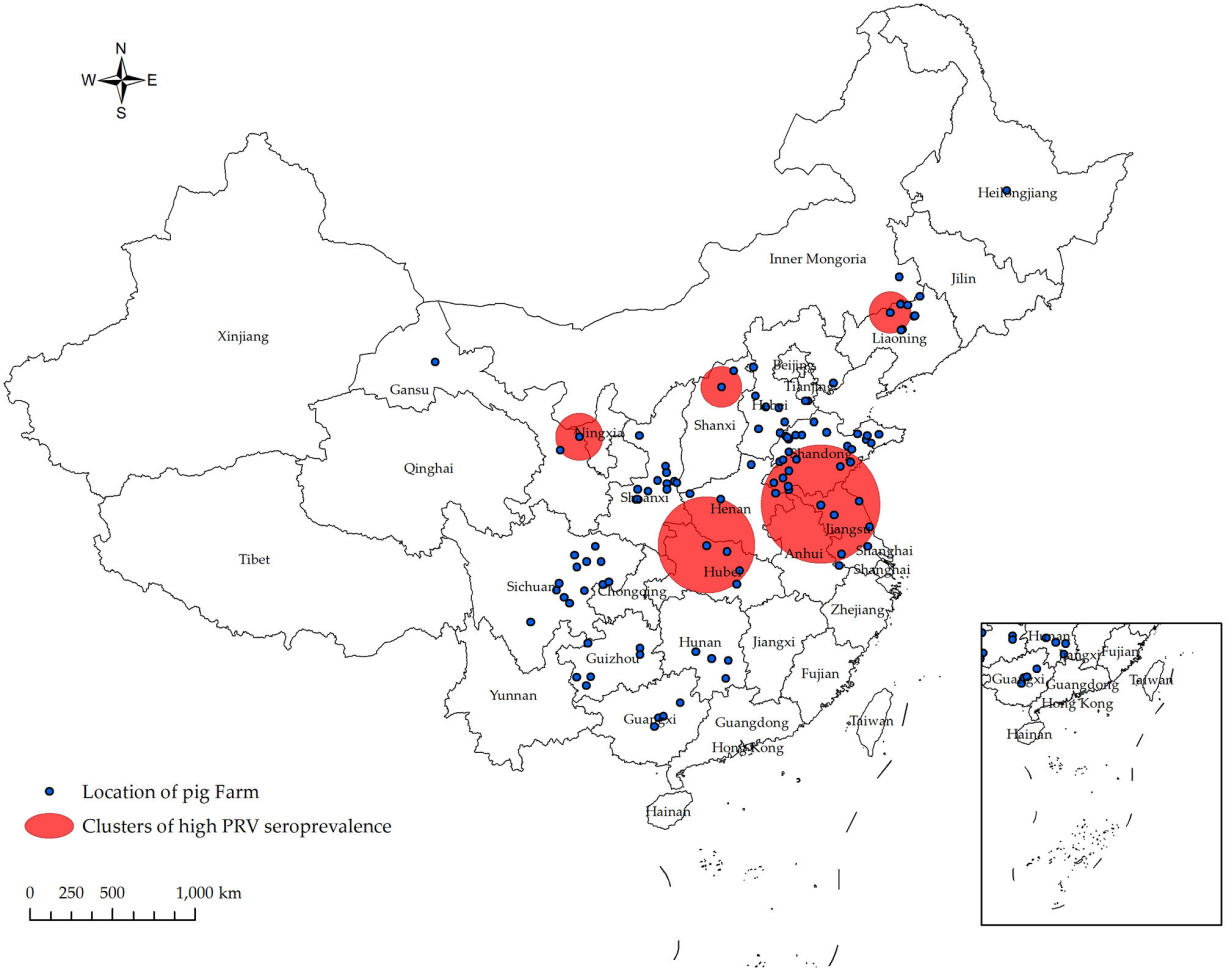
Significant spatial clusters (*p* < 0.05) of high PRV gE seroprevalence were observed in China from January 2022 to December 2022 with a maximum window size of 50% of the population at risk. Using SaTScan 10.1 software, the PRV gE antibody status of 556 pig farms from 106 companies was analyzed. The Bernoulli model was used to predict the temporal and spatial distribution of PRV gE serum high prevalence aggregation. From January 2022 to December 2022, five populations of PRV gE with a high seroprevalence were identified in China.

**Figure 5:**
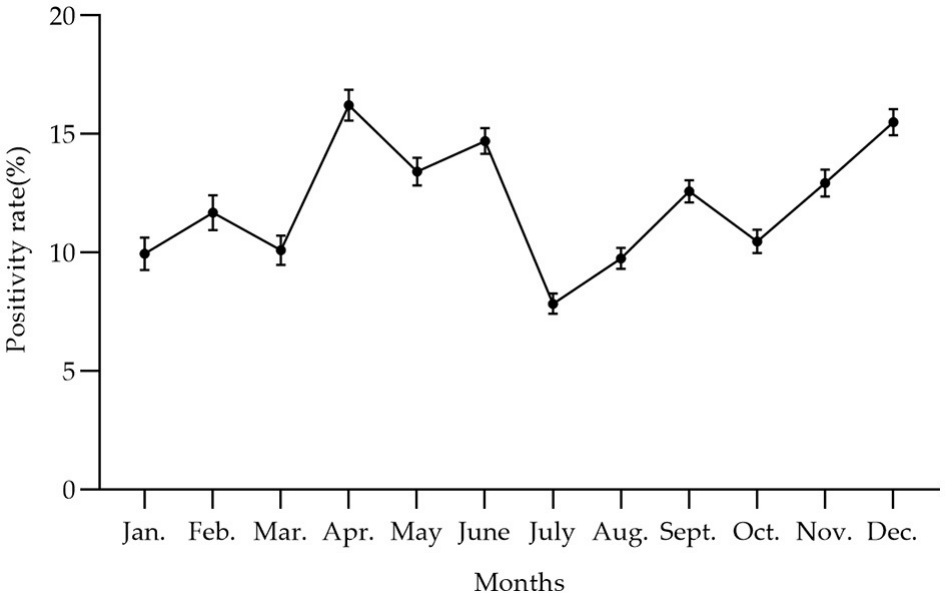
The trend of PRV gE antibody positivity rate at the animal level varied by month from January 2022 to December 2022 in China, with the peak of PRV gE seroprevalence occurring from April to June. The positivity rates of the PRV gE antibody from January to December were 9.96, 11.69, 10.11, 16.23, 13.42, 14.71, 7.84, 9.76, 12.59, 10.48, 12.94, and 15.51%, respectively.

In Figure 4 and Table 4, the survey results show the high serum prevalence of five clusters of PRV gE found in China from January 2022 to December 2022. The first cluster was located at latitude 42.313505° N, longitude 121.849035° E, with a radius of 92.88 km. It spanned from June 1, 2022, to November 30, 2022, and had a relative risk value of 6.50 and a log-likelihood ratio (LLR) value of 2,037.92 (*p* < 0.001). The second cluster was located at latitude 32.183738° N, longitude 111.832792° E, with a radius of 247.57 km. It occurred from December 1, 2022, to December 31, 2022, with a relative risk value of 6.54 and an LLR value of 1,675.08 (*p* < 0.001). The third cluster is the first large area with a radius of 297.86 km, located at latitude 34.040922° N and longitude 118.058937° E. It spans from May 1, 2022, to October 31. The relative risk value is 2.97, and the LLR value is 927.48 (*p* < 0.001). The fourth cluster is situated at coordinates 39.226308° N and 112.643375° E, covering an area with a radius of 95.62 km. The cluster encompasses the time period from April 1, 2022, to September 30, 2022. The relative risk value was found to be 7.07, while the LLR value was calculated to be 927.34 (*p* < 0.001). The fifth cluster was identified at geographical coordinates 37.074554° N, 104.895751° E, encompassing an area with a radius of 113.76 km. The time range of the cluster spans from August 1, 2022, to December 31, 2022. The relative risk value was 2.94, and the likelihood ratio value was 898.77 (*p* < 0.001). Meanwhile, Figure 5 and Table 5 show a high seropositivity rate of PRV gE during the period from April 2022 to June 2022. The relative risk value was 1.27 and the LLR value was 120.88 (*p* < 0.01).

**Table 4:** Spatial-temporal clusters of PRV gE seroprevalence in China in 2022.

**Cluster**	**Coordinates**	**Cluster radius (km)**	**Time range (yr/mo/day)**	**Relative risk**	**Log likelihood ratio**	***P*-value**
1	42.313505 N, 121.849035 E	92.88	2022/6/1–2022/11/30	6.5	2,037.92	<10^−17^
2	32.183738 N, 111.832792 E	247.57	2022/12/1–2022/12/31	6.54	1,675.08	<10^−17^
3	34.040922 N, 118.058937 E	297.86	2022/5/1–2022/10/31	2.97	927.48	<10^−17^
4	39.226308 N, 112.643375 E	95.62	2022/4/1–2022/9/30	7.07	927.34	<10^−17^
5	37.074554 N, 104.895751 E	113.76	2022/8/1–2022/12/31	2.94	898.77	<10^−17^

**Table 5:** The time period of PRV gE seroprevalence in China in 2022.

**Cluster**	**Time frame (yr/mo)**	**Relative risk**	**Log likelihood ratio**	***P-*value**
1	2022/4/−2022/6	1.27	120.88	0.001

## 4 Discussion

The PRV-gE deletion vaccine has demonstrated efficacy in preventing PRV and has been used in both large-scale and small-scale Chinese farms for an extended period (25). Consequently, the presence of PRV-gE antibodies serves as an indicator of the prevalence of wild-type PRV strains. Previous investigations have indicated that the prevalence of PRV-gE antibodies in pig farms in China has exhibited a gradual increase from 2011 to 2016, followed by a subsequent decline from 2016 to 2021. Notably, the overall positive rate of PRV-gE antibodies has remained at ~20% over the past 2 years (26–35).

In this study, the objective was to evaluate the existing seroprevalence of wild-type PRV gE in China by collecting blood samples from pigs from January 2022 to December 2022. We conducted a series of tests to detect antibodies against PRV gE. This study encompassed an analysis of 161,880 serum samples obtained from 556 pig farms of different scales (large, medium, and small) located in 16 provinces throughout China. We conducted an analysis on the spatiotemporal distribution characteristics of the infection rate of wild-type PRV strains in China, and successfully identified the associated related factors.

In the conducted survey, the collective serum samples exhibited an overall positive rate of PRV gE antibodies at 12.36%, while the positive rate in farms was significantly higher at 46.22%. There are significant variations in the rates of antibody positivity and farm positivity among pig herds in different provinces. This suggests that the prevalence of pseudorabies wild virus infection varies across regions. Chen et al. (33) conducted an analysis on a total of 35,796 serum samples collected in Henan Province between 2019 and 2021. They observed a decline in the prevalence of PRV gE antibodies from 25 to 16.69%. Similarly, Zhou et al. (27) found that the positive rate in Heilongjiang Province from 2013 to 2018 was 16.3%. This study revealed that the prevalence of positivity in Henan and Heilongjiang provinces was 32.96% (1,511/4,584, 95% CI 30.59–35.33%) and 21.71% (2,984/13,747, 95% CI 21.02–22.40%), respectively. These rates indicate a higher prevalence compared to the study conducted by Zhou et al. (27) and Chen et al. (33). This phenomenon could potentially be associated with variables such as the quantity of serum samples collected and the number of pig farms included in the study. The serological survey of PRV in Hunan Province, conducted by Lin et al. from 2016 to 2020, revealed a PRV-gE antibody positivity rate of 23.55% (4,271/18,138, 95% CI 22.9–24.2%) (28). In the present study, the prevalence of PRV gE antibody positivity in Hunan Province was found to be 13.56% (236/1,740, 95% CI 11.95–15.17%), which is significantly lower than the findings reported by Lin et al. (28) in their survey. Similarly, in previous reports, the serum positive rate in Shandong Province from 2013 to 2016 was recorded at 57.8% (2,909 out of 5,033) (26). In the case of Tianjin, the positive rate from 2010 to 2018 was reported as 46.70% (34). Lastly, Hebei Province recorded a positivity rate of 46.27% from 2017 to 2018 (29). In the current investigation, the prevalence rates recorded in Shandong (11.36%), Tianjin (0.19%), and Hebei (17.04%) were determined to be lower compared to previously documented rates. This phenomenon can be ascribed to alterations in the distribution of Chinese pig populations resulting from the African swine fever crisis. The continuous decline in the serum positivity rate of PRV gE antibodies indicates that significant advancements have been made in the country's efforts to manage the transmission of wild pseudorabies virus infection.

Sows infected with PRV have the ability to transmit maternal antibodies to their offspring via colostrum, which can persist in piglets for a duration of 12–14 weeks (36). The present investigation revealed that the seroprevalence was significantly higher in piglets (21.86%) compared to sows (19.75%). However, the odds ratio (OR) value of piglets relative to sow infection was 1.04 (0.98–1.10), and the *p*-value was >0.05. This difference may be attributed to the presence of positive maternal antibodies against PRV-gE in sows. Notably, the seropositive rate of pigs declined gradually with age, from nurseries to gilts, indicating the effectiveness of current strategies in preventing wild-type PRV strains. While the seropositive rate of sows is still high, there is a need to develop more effective strategies in the future to specifically target sows and pre-wean piglets.

From a geographical perspective, the rates of serum positivity for PRV are higher in the Northeast and Central South regions compared to other regions. The Northeast region has the highest likelihood of PRV infection, while the Southwest region has the lowest likelihood. This result is slightly different from the research findings of Liu et al. (37). A study on the prevalence of PRV in Chinese pig farms from 2013 to 2016 showed that in 2016, the PRV-gE antibody serum positive rate was highest, and the risk was highest in North China, followed by the central and southern regions. It may be related to the variations in pig distribution across different regions and the increased frequency of updates on pig populations following the outbreak of African swine fever diseases. The positive rates of PRV gE antibodies in the six regions studied showed significant differences (*p* < 0.01), indicating variations in PRV wild-type virus infection rates across the country.

On the spatiotemporal analysis of PRV serum positivity rate, Allepuz et al. (38) conducted a study to analyze the spatial distribution of PRV incidence rate among pig farms in Catalonia, Spain, from 2003 to 2007. The study aimed to determine the clustering of PRV infection in both sows and fattening pig farms. Berke et al. (39) detected the spatial aggregation of two PRV infections in Germany, with radii of 2.6 and 1.7 km, and relative risk values of 2.4 and 3.3, respectively. Zhao et al. (22) detected five significant high PRV gE seroprevalence groups in China for the first time in their data from 2017 to 2021. However, research on PRV spatiotemporal clustering in China is still relatively limited. As Zhao et al. (40) stated in his study, conducting spatiotemporal analysis of PRRSV infections can assist in identifying clusters with high PRRSV prevalence and examining the evolving patterns of PRRSV infections. This analysis can aid policy makers in developing more accurate and cost-effective interventions for future PRRSV control in China. Similarly, conducting spatiotemporal analysis of pseudorabies infections is also significant in this regard.

From a quarterly perspective, the seroprevalence rate of PRV-gE antibodies fluctuated throughout the year, reaching its peak in the second quarter (Q2), and Q2 has the highest risk of infection. This finding is consistent with a previous report (33). This could be attributed to the significant temperature variation between day and night during this period.

## 5 Conclusion

We conducted a seroepidemiologic survey for wild type PRV gE in China from January 2022 to December 2022. During this period, we collected 161,880 blood samples from 556 swine farms belonging to 106 companies across 16 provinces. These samples were then tested for PRV gE antibodies using ELISA. At the animal level and the farm level, the overall seroprevalence of PRV gE was 12.36% (95% CI, 12.20–12.52%) and 46.22% (95% CI, 42.08–50.37%), respectively. In addition, the seroprevalence of PRV varied significantly among provinces and herds. We conducted a comprehensive study utilizing logistic regression analysis to identify the related factors associated with Pseudorabies virus (PRV) serologic status on farms. These factors include the geographic location of the farm, herd type, and season. We identified five distinct PRV gE seroprevalence clusters in China from April 1, 2022, to December 31, 2022. Additionally, we found that the months with higher PRV gE seropositivity in China in 2022 were from April to June. Our findings serve as a valuable addition to existing research on the seroprevalence, related factors, and temporal clustering of PRV gE in China. Furthermore, our study provides a reference point for the development of effective strategies for the prevention and control of pseudorabies and wild virus outbreaks.

## Data availability statement

The original contributions presented in the study are included in the article/supplementary material, further inquiries can be directed to the corresponding author.

## Ethics statement

The animal studies were approved by the Animal Welfare Guidelines of the World Organization for Animal Health. The studies were conducted in accordance with the local legislation and institutional requirements. Written informed consent was obtained from the owners for the participation of their animals in this study.

## Author contributions

WG: Conceptualization, Data curation, Investigation, Software, Visualization, Writing—original draft. XJ: Data curation, Investigation, Software, Visualization, Writing—original draft. ZH: Validation, Writing—review & editing. QW: Data curation, Writing—original draft. YS: Data curation, Writing—original draft. XT: Investigation, Writing—original draft. MQ: Investigation, Writing—original draft. JZ: Writing—review & editing. YL: Validation, Writing—review & editing. XL: Conceptualization, Formal analysis, Funding acquisition, Methodology, Project administration, Resources, Supervision, Validation, Writing—review & editing.
